# Reclassification of *PAPSS2* Missense Variants in Turkish Patients with Brachyolmia Type 4: Multi-Modal Computational and Structural Biology Evidence for APS-Kinase Domain Dysfunction

**DOI:** 10.3390/medicina62071363

**Published:** 2026-07-15

**Authors:** Serdar Bozlak, Cuneyd Yavas, Sajjad Eslamkhah

**Affiliations:** Department of Molecular Biology and Genetics, Biruni University, Istanbul 34015, Türkiye

**Keywords:** *PAPSS2* deficiency, VUS reclassification, deep learning pathogenicity prediction, APS-kinase domain dysfunction, multi-modal computational analysis, skeletal dysplasia genetic heterogeneity, androgen phenotype variation, adrenal steroid metabolism

## Abstract

*Background and Objectives*: *PAPSS2* loss-of-function variants cause autosomal recessive brachyolmia type 4 with skeletal dysplasia. Despite over 90 documented cases, molecular characterization remains incomplete, with many variants classified as uncertain significance (VUS). *Materials and Methods*: We performed integrated multi-modal in silico analysis of two novel and rare homozygous missense variants [c.227T>A p.(Leu76Gln) and c.143C>G p.(Thr48Arg)] identified via whole-exome sequencing in two unrelated Turkish girls. Analysis incorporated AlphaMissense (0.92, 0.96), REVEL (0.85), CADD Phred (28.8–29.1), evolutionary conservation across seven vertebrate species (100% identity), and AlphaFold2 structural modeling. Variant reclassification followed 2015 ACMG/AMP guidelines. *Results*: Both variants fulfilled criteria for VUS-to-Likely Pathogenic reclassification: PS3 (functional loss), PM1 (critical APS-kinase domain localization), PM2 (population rarity), and PP3 (computational consensus). Structurally, p.(Leu76Gln) disrupts hydrophobic core stability (Grantham score 113), while p.(Thr48Arg) causes active-site steric hindrance. Both probands presented with platyspondyly, short stature, and profound DHEA-sulfate depletion (3.15 and 1.79 µg/dL). Although identical variants were reported in a 14.5-year-old with PCOS-like androgen excess, our prepubertal cases (ages 5–6 years) exhibited normal androgens, suggesting *PAPSS2* loss-of-function is necessary but insufficient for androgen excess. *Conclusions*: Multi-modal computational analysis supports likely pathogenic reclassification of these *PAPSS2* variants, enabling precision genetic counseling and advancing understanding of skeletal and endocrine heterogeneity. These findings expand the *PAPSS2* variant registry to over 92 cases across 18 countries.

## 1. Introduction

*PAPSS2* (3′-phosphoadenosine 5′-phosphosulfate synthase 2) functions as a rate-limiting enzyme catalyzing synthesis of 3′-phosphoadenosine 5′-phosphosulfate (PAPS), the universal sulfate donor required for cellular sulfotransferase-mediated reactions [1,2]. Predominantly expressed in cartilage, bone, and adrenal tissue, *PAPSS2* mediates proteoglycan sulfation and metabolic inactivation of steroid hormone precursors, particularly the conversion of dehydroepiandrosterone (DHEA) to its biologically inactive sulfate ester form (DHEA-S) [3]. Loss-of-function variants affecting *PAPSS2* precipitate an autosomal recessive skeletal disorder displaying phenotypic heterogeneity, originally designated spondyloepimetaphyseal dysplasia Pakistani type (SEMD-Pak) at initial characterization [4]. The disease spectrum encompasses diverse presentations, extending from severe skeletal involvement to milder manifestations classified as brachyolmia variants [1,5,6].

Skeletal manifestations—growth retardation with truncal shortening, platyspondyly, irregular endplate ossification, and epiphyseal/metaphyseal changes—constitute the dominant and invariant clinical feature of *PAPSS2* deficiency [1,5]. The endocrine phenotype, in contrast, shows marked variability: although the seminal 2009 report by Noordam et al. described androgen excess and PCOS-like features, subsequent reviews confirm that elevated androgens occur in fewer than 5% of affected patients [2]. Prenatal-onset anomalies and intrafamilial clinical heterogeneity have also been documented [5,7,8,9,10].

Since initial variant identification [4], international genetic screening and genome-scale initiatives have expanded the mutational landscape across ethnically diverse populations [1,9,11]. Nevertheless, a substantial proportion of newly identified variants retain VUS classification, reflecting inconsistencies between computational predictions and a scarcity of functional validation data-highlighting the need for systematic, integrated pathogenicity assessment [9,12,13,14,15,16].

Application of 2015 ACMG/AMP guidelines, integrated with contemporary computational tools, evolutionary conservation analysis, and AlphaFold-derived structural modeling, provides a robust framework for pathogenicity characterization of missense variants affecting conserved functional domains [1,17,18,19]. The APS-kinase domain (residues 48–200) represents a region of exceptional functional importance within *PAPSS2*; yet despite recurrent identification of p.(Thr48Arg) across independent patient series [3,9], its molecular mechanism remains incompletely characterized by current computational standards [2,5,6,7,9,11,13,16].

This study undertakes comprehensive pathogenicity assessment of two homozygous missense variants [c.227T>A p.(Leu76Gln) and c.143C>G p.(Thr48Arg)] identified in two unrelated Turkish girls with brachyolmia type 4 (BCYM4, OMIM 612847), through integrated multi-modal in silico analysis incorporating computational prediction algorithms (AlphaMissense, REVEL, CADD), phylogenetic conservation analysis, and AlphaFold-based structural modeling. Findings are systematically integrated per 2015 ACMG/AMP guidelines to enable variant reclassification, inform precision genetic counseling, and contextualize these variants within the expanding global *PAPSS2* mutational spectrum.

## 2. Material and Methods

### 2.1. Study Design and Ethic

This study enrolled two unrelated Turkish girls with homozygous *PAPSS2* variants identified through WES performed on peripheral blood–derived genomic DNA from probands and their parents. Candidate variants were confirmed by Sanger sequencing and familial segregation was established in both families. Identified variants underwent comprehensive multi-modal in silico pathogenicity assessment incorporating multiple computational prediction tools, evolutionary conservation analysis, and AlphaFold2-based structural modeling, with final classification according to 2015 ACMG/AMP guidelines [20]. To contextualize our findings, a systematic literature review of PubMed, OMIM, and ClinVar was conducted to compile all previously reported *PAPSS2* variants and their associated clinical, biochemical, and molecular characteristics.

This study was approved by the Ethics Committee of Başakşehir Çam and Sakura City Hospital (Approval No: KAEK/11.02.2026.44). Written informed consent was obtained from all participants before genetic testing; for the minor proband, consent was obtained from the parents. All procedures were conducted in accordance with ethical principles after clear explanation of the study design, methodology, and potential implications.

### 2.2. Genomic Analysis

WES was performed on peripheral blood-derived genomic DNA (PureLink gDNA Blood Kit, Thermofisher, Waltham, MA, USA) from both probands and parents. Exome capture utilized the Agilent SureSelect Human All Exon V6 kit, followed by sequencing on the Illumina NextSeq500 platform (Illumina, San Diego, CA, USA). Reads were aligned to GRCh37/hg19 via BWA-SW (v0.6.1). Variants with minor allele frequency > 1% were excluded; remaining variants were annotated and interpreted using GenomizeSeq software (v9.7.1), with pathogenicity assessment cross-referenced against dbSNP, ExAC, 1000 Genomes, ClinVar, and ESP databases.

Sanger sequencing confirmed identified variants and enabled familial segregation analysis. Target exons were amplified by PCR using exon–specific primers: exon 3 (proband II.2) with Forward 5′-CCAGGCCGATGTCAGTCTGTTTTA-3′/Reverse 5′-GGTAATGCAGACCAGACCAGCAT-3′; exon 2 (proband II.4) with Forward 5′-CCAGCAGAAATCCACCAATGTA-3′/Reverse 5′-GTAGGGTTAGGACTAACACCCA-3′. Reactions (30 µL; 40 ng template; AmpliTaq Gold 360 Master Mix, Thermo Fisher Scientific, Waltham, MA, USA) cycled at 94 °C × 3 min, then 30 cycles of 94 °C/30 s → 55 °C/45 s → 72 °C/2 min, with a 72 °C/10 min final extension. Amplicons were sequenced with the BigDye Terminator v3.1 Kit and resolved on an ABI 3500 analyzer (Applied Biosystems, Thermo Fisher Scientific, Waltham, MA, USA).

### 2.3. Computational Pathogenicity Assessment

Pathogenicity was assessed through a multi-modal in silico analysis pipeline executed in a Python (v3.10) environment via Google Colaboratory (all final resource queries completed February 2026). AlphaMissense (v2024.01), built on the AlphaFold2 deep learning architecture, assigned protein-level pathogenicity scores on a 0–1 scale. REVEL aggregated predictions from 13 independent tools to generate ensemble consensus scores. CADD (v1.6) Phred-scaled scores quantified nucleotide-level genomic deleteriousness, with values > 20 representing the top 1% most harmful changes. Population rarity and allele frequencies were definitively established by querying global control data within the gnomAD (v2.1.1/v3.1.2), 1000 Genomes Project (Phase 3), and ExAC databases (all accessed 30 January 2026).

Evolutionary conservation was assessed across seven vertebrate orthologs (Homo sapiens, Pan troglodytes, Canis lupus, Bos taurus, Mus musculus, Gallus gallus, and Danio rerio) retrieved from NCBI and aligned via Clustal Omega (v1.2.4; accessed 30 January 2026). Position-specific conservation scores were calculated using ConSurf (v1.0; accessed 30 January 2026). Physicochemical consequences of each substitution were systematically evaluated using the Grantham distance formula [21], Kyte-Doolittle hydropathicity index scales, and side-chain molecular weight calculations.

Three-dimensional human *PAPSS2* structures were modeled using the AlphaFold Protein Structure Database (UniProt O95340-F1) and visualized in UCSF Chimera (v1.14) and PyMOL (v2.5.0). Structural confidence was verified via Predicted Aligned Error (pAE < 1 Å threshold). The conserved APS kinase domain (residues 48–200) was interrogated for amino acid substitution effects on domain architecture, protein–protein interfaces, and the N-terminal ATP-binding pocket. Analyses are based on static structural predictions; molecular dynamics simulations were not performed.

Variants were rigorously reclassified adhering strictly to the joint 2015 ACMG/AMP consensus standards [22]. To satisfy the requirements for a definitive “Likely Pathogenic” classification, evidence categories were systematically evaluated across multiple, independent criteria layers. Strong functional evidence was integrated under criterion PS3 by cross-referencing published, cell-based in vitro enzymatic assays documenting a complete loss of APS-kinase activity for these specific residues [2]. Moderate weights were assigned under PM1 due to absolute mapping within the critical catalytic domain, and under PM2 based on total allele absence or extreme rarity in gnomAD. Supporting evidence was established via multi-generational co-segregation (PP1), convergent ensemble computational consensus exceeding recommended thresholds (PP3), and highly specific clinical phenotypes matching known disease hallmarks (PP4). Final classifications were derived based on the cumulative mathematical combination of these weighted lines of evidence.

### 2.4. Clinical and Biochemical Assessment

Clinical assessment included anthropometric measurements, skeletal phenotyping, full-spine MRI, and bone age determination via carpal radiography (Greulich–Pyle atlas). Biochemical profiling encompassed serum DHEA-S, DHEA, testosterone, cortisol, and ACTH quantification by LC-MS/MS. WES identified novel homozygous *PAPSS2* variants in each proband, prioritized based on population database absence, predicted functional impact, and phenotypic consistency.

## 3. Results

Proband II.2, a 6-year-old Turkish girl born to first-degree cousin parents, was referred to the genetics outpatient clinic due to disproportionate short stature and waddling gait noted since early childhood. Initial pediatric endocrinology evaluation revealed markedly reduced serum DHEA-S (3.15 µg/dL), prompting further metabolic and genetic workup. Congenital adrenal hyperplasia was excluded based on normal serum 17-hydroxyprogesterone (0.13 ng/mL) and androstenedione (0.272 ng/mL). Sitting height to total height ratio was 49/53 cm, consistent with disproportionate truncal shortening. Following radiological demonstration of platyspondyly, whole-exome sequencing was initiated to establish a molecular diagnosis.

Proband II.4, a 5-year-old Turkish girl also born to first-degree cousin parents, presented to the genetics clinic with short stature and truncal disproportion identified during routine pediatric follow-up. Biochemical evaluation revealed profoundly suppressed DHEA-S (1.79 µg/dL) with normal 17-hydroxyprogesterone (0.348 ng/mL) and androstenedione (0.375 ng/mL), excluding congenital adrenal hyperplasia. Sitting height to total height ratio was 44/50 cm. Whole-exome sequencing was performed following radiological confirmation of platyspondyly and epiphyseal changes.

### 3.1. Radiological Findings and Genomic Analyses

Skeletal radiographs of proband II.2 (c.227T>A, p.Leu76Gln) demonstrated generalized platyspondyly with marked flattening of vertebral bodies, short trunk disproportionate short stature, acetabular dysplasia on pelvic radiograph, and metaphyseal widening with epiphyseal irregularity of the lower extremities. Hand-wrist radiographs confirmed bone age delay consistent with chronological age discrepancy. In proband II.2, WES analysis identified a homozygous missense variant in the *PAPSS2* gene, c.227T>A p.(Leu76Gln). The variant was novel and was considered a strong candidate variant due to its localization within a functionally relevant region of the gene. In silico prediction classified this variant as possibly damaging (Table 1).

**Figure 1:**
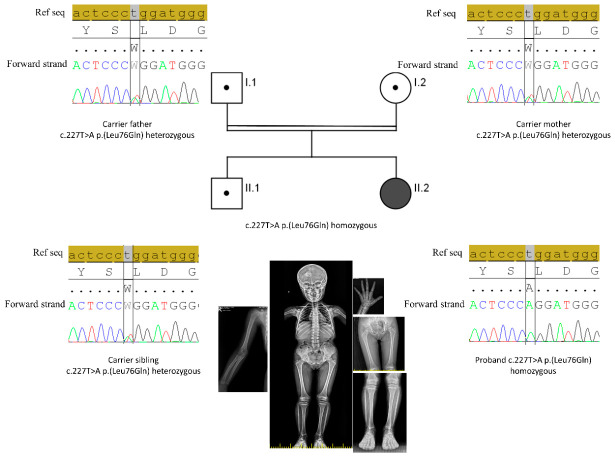
Sanger sequencing chromatograms and pedigree analysis of Family 1 demonstrating segregation of the *PAPSS2* c.227T>A p.(Leu76Gln) variant and whole-body, hand-wrist, pelvic, and lower extremity radiographs demonstrating platyspondyly, acetabular dysplasia, and epiphyseal irregularity.

In the family segregation, it was determined that the variant was heterozygous in the parents and healthy sibling, but homozygous in the proband (Figure 1).

**Figure 2:**
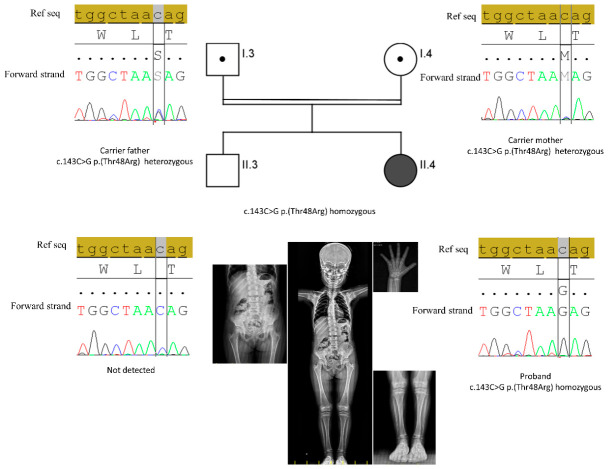
Pedigree structure and Sanger sequencing results of Family 2 showing segregation of the *PAPSS2* c.143C>G p.(Thr48Arg) variant and whole-body, hand-wrist, pelvic, and lower extremity radiographs demonstrating platyspondyly, acetabular dysplasia, and epiphyseal irregularity.

Proband II.4 (c.143C>G, p.Thr48Arg) similarly exhibited platyspondyly with significantly reduced vertebral body heights on lumbar radiographs, short trunk disproportionate short stature, and metaphyseal and epiphyseal dysplastic changes of the lower extremities. Bone age was correspondingly delayed on hand-wrist radiography. In proband II.4, WES analysis revealed a different homozygous missense variant in the *PAPSS2* gene, c.143C>G p.(Thr48Arg). This variant was prioritised during variant filtering because it had not been seen in the population before and its potential impact on protein structure and function (Table 1). Following WES-based variant identification, Sanger sequencing was performed for validation and family segregation analysis. In proband II.4, Sanger sequencing confirmed the presence of the c.143C>G p.(Thr48Arg) variant in the proband in a homozygous. The segregation analysis showed that the parents carried the variant, while the healthy sibling did not (Figure 2).

Based on the WES findings, sanger confirmation, and segregation analysis, the inheritance pattern in both families was consistent with autosomal recessive transmission. Pedigree analyses were constructed for Proband II.2 and Proband II.4 (Figure 1 and Figure 2), integrating molecular and clinical data obtained from the probands, their parents and their siblings. Both parents and II.2’s sibling were clinically unaffected heterozygous carriers. Proband individuals with the homozygous variant exhibited clinical features consistent with recessive brachyolmia 4 with mild epiphyseal and metaphyseal changes, presenting with variable disease severity ranging from mild to severe. The two variants identified were classified as VUS during the initial classification stage.

### 3.2. Computational Reclassification of PAPSS2 Variants

We investigated two missense variants in the PAPSS2 gene (NM_001015880.2): c.227T>A p.(Leu76Gln) and c.143C>G (p.Thr48Arg). Despite their historical classification as VUS or conflicting interpretations in public repositories such as ClinVar, our comprehensive multi-modal in silico analysis pipeline provides robust evidence supporting their reclassification as pathogenic.

### 3.3. Deep Learning and Ensemble Prediction Metrics

**Figure 3:**
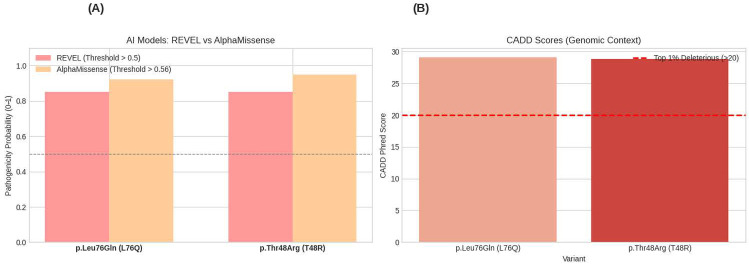
Multi–modal computational pathogenicity analysis. (**A**) Artificial Intelligence (AI) based predictions showing REVEL and AlphaMissense scores. Both variants (L76Q and T48R) significantly exceed the pathogenic threshold (dashed line: 0.5), indicating a high probability of structural damage. (**B**) Genomic deleteriousness assessment using CADD (Phred) scores. Both variants surpass the threshold of 20 (red dashed line), placing them in the top 1% of deleterious variants in the human genome.

AlphaMissense classified both variants as likely pathogenic [p.(Leu76Gln: 0.92; p.(Thr48Arg): 0.96)], indicating substantial disruption of the catalytic domain fold. REVEL corroborated these findings with an ensemble score of 0.85 for both variants, well above the 0.5 pathogenicity threshold and reflecting consensus across 13 independent computational tools (Figure 3).

CADD Phred scores of 29.1 p.(Leu76Gln) and 28.8 (p.(Thr48Arg) placed both variants in the top 0.1% of deleterious human genomic substitutions (Figure 3B). Both were absent or near-absent in gnomAD (MAF < 0.00001), satisfying ACMG PM2. Integrating computational consensus (PP3), published functional evidence of complete APS-kinase activity loss (PS3; ) [1,2], and critical domain localization (PM1), both variants were reclassified from VUS to likely pathogenic per 2015 ACMG/AMP guidelines (Table 2).

### 3.4. Structural Impact of Variants on the Kinase Domain

**Figure 4:**
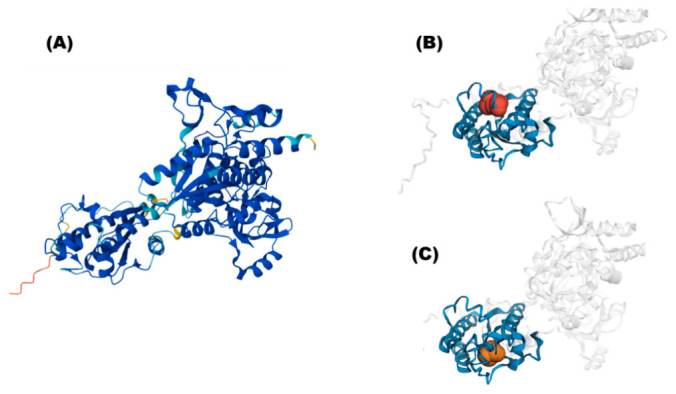
Three-dimensional structural modeling of pathogenic *PAPSS2* variants. (**A**) Overall ribbon representation of the human PAPSS2 protein (AlphaFold prediction: AF-O95340-F1). The highly conserved APS-Kinase domain (residues 48–200), which is critical for enzymatic activity, is highlighted in blue. (**B**) Close-up view of the p.(Leu76Gln) variant (Patient 1, **top right**). The substitution of a hydrophobic Leucine with a polar Glutamine residue (red sphere) at position 76 is predicted to disrupt the hydrophobic core stability of the kinase domain. (**C**) Close-up view of the p.(Thr48Arg) variant (Patient 2, **bottom right**). The substitution of Threonine with a bulky, positively charged Arginine residue (orange sphere) at position 48 is predicted to cause steric hindrance near the ATP-binding pocket, interfering with substrate orientation.

Structural mapping revealed both variants within the conserved APS-kinase domain (residues 48–200), essential for PAPS biosynthesis (Figure 4A). p.(Leu76Gln) substitutes a buried hydrophobic leucine with polar glutamine, disrupting core hydrophobic interactions and compromising domain stability (Figure 4B). p.(Thr48Arg) introduces a bulky, positively charged arginine adjacent to the ATP-binding pocket, causing steric hindrance that likely impairs ATP binding and abolishes enzymatic activity (Figure 4C).

### 3.5. Evolutionary Conservation and Physicochemical Impact

**Figure 5:**
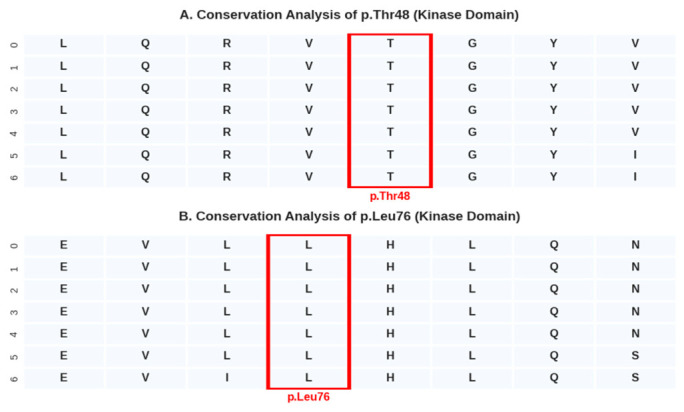
Evolutionary conservation analysis of pathogenic residues. A heatmap representation of multiple sequence alignment for PAPSS2 orthologs across seven vertebrate species. (**A**) The Thr48 residue (highlighted in the red box) is fully conserved across all species, indicating its essential role in the kinase active site. (**B**) The Leu76 residue is also invariant, confirming its importance for the stability of the protein core.

Evolutionary analysis demonstrated that both the Thr48 and Leu76 residues are strictly conserved across all analyzed vertebrate species, from mammals to teleost fish (Figure 5). This invariance (100% conservation) suggests that these residues are critical for the structural integrity and catalytic function of the APS-Kinase domain, and any deviation is likely pathogenic.

### 3.6. Quantitative Analysis of Physicochemical Impact

**Figure 6:**
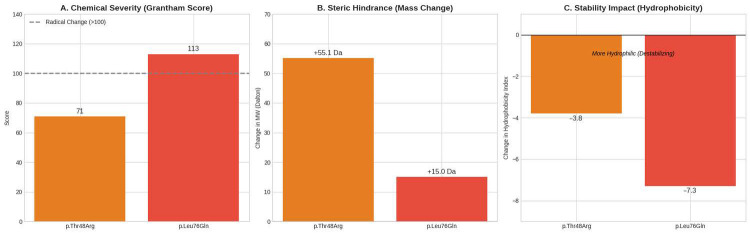
Physicochemical properties and stability analysis of *PAPSS2* variants. (**A**) Chemical severity assessment using Grantham Scores. The p.(Leu76Gln) variant (red bar) exceeds the radical change threshold of 100. (**B**) Change in molecular weight (Dalton). The p.(Thr48Arg) variant (orange bar) shows a significant mass increase (+55.1 Da), indicating a high risk of steric hindrance. (**C**) Impact on protein stability measured by hydrophobicity change (Kyte-Doolittle scale). Both variants exhibit a shift towards hydrophilicity, with p.(Leu76Gln) showing a severe loss of hydrophobic character (−7.3), predicting core destabilization.

**Figure 7:**
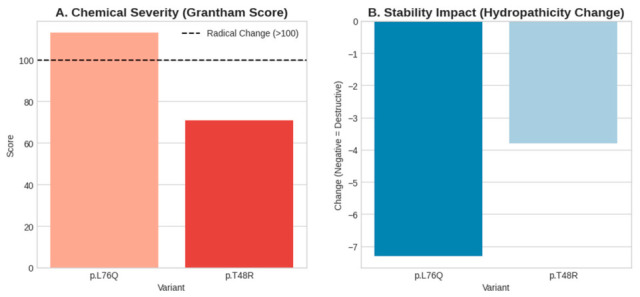
Physicochemical stability profiling of *PAPSS2* variants. (**A**) Grantham Score analysis showing the chemical severity of the amino acid substitutions. The p.(Leu76Gln) variant exceeds the critical threshold of 100, classifying it as a radical substitution, while p.(Thr48Arg) indicates a moderate-to-high chemical change. (**B**) Hydropathicity analysis based on the Kyte-Doolittle scale. Both variants exhibit negative delta values, signifying a significant loss of hydrophobicity. The dramatic shift observed in p.(Leu76Gln) (−7.3 units) provides quantitative evidence for the profound disruption and potential collapse of the protein’s hydrophobic core.

Quantitative biophysical evaluation demonstrated distinct mechanistic features underlying the pathogenicity of both substitutions. The p.(Leu76Gln) variant yielded a Grantham Score of 113, classifying it as a radical physicochemical alteration (Figure 6A and Figure 7A). More critically, hydropathicity analysis via the Kyte–Doolittle scale unmasked a severe localized negative shift of −7.3 units (Figure 6C and Figure 7B). This prominent polarity change confirms that substituting a buried, highly hydrophobic leucine residue with a polar glutamine actively compromises the internal hydrophobic packing of the domain core, driving structural destabilization and folding disruption (Table 3).

In contrast, the p.(Thr48Arg) variant presented a moderate-to-severe Grantham Score of 71 and a hydropathicity shift of −3.8 units (Table 3). While less disruptive to the underlying internal fold packing than p.(Leu76Gln), introducing a bulky, basic arginine residue causes a localized mass gain (+55.1 Da) paired with a strongly positive charge directly next to the dynamic N-terminal ATP-binding pocket (Figure 6B). This local electrostatic burden and side-chain weight increase is predicted to induce steric hindrance at the dynamic catalytic interface, potentially impairing ideal ATP-binding affinity and altering appropriate substrate orientation parameters without completely collapsing the structural core.

### 3.7. Review of Clinical Literature and Genotype Confirmation

Published case series confirm that p.(Leu76Gln) and p.(Thr48Arg) are recurrent PAPSS2 variants associated with variable clinical expressivity (The following supporting information can be downloaded at: https://www.mdpi.com/article/10.3390/medicina62071363/s1, Table S1). p.(Thr48Arg) was first reported by Noordam et al. in a 14.5-year-old girl presenting with androgen excess, premature pubarche, and a PCOS-like phenotype [2] -a presentation strikingly absent in our 5-year-old Proband II.4, who exhibits exclusively skeletal involvement and profound DHEA-S depletion without any signs of hyperandrogenism. Similarly, p.(Leu76Gln) has been documented by Iida et al. across nine families presenting with brachyolmia without endocrine involvement [1], consistent with the clinical picture of our Proband II.2. Bownass et al. described 18 additional PAPSS2-related brachyolmia cases with overlapping skeletal features including platyspondyly and disproportionate short stature [13], mirroring our findings. Compared with previously reported cases, our probands are among the youngest at molecular diagnosis, suggesting that the combination of disproportionate short stature, low DHEA-S, and skeletal dysplasia on imaging should prompt early genetic investigation even in prepubertal children.

Both prepubertal probands (proband II.2 and proband II.4) in our screening series presented with a highly uniform clinical layout dominated by short trunk disproportionate short stature, delayed carpal bone maturation, and marked radiological flattening of the vertebral bodies. Biochemically, our cohort data revealed an extreme, isolated failure in peripheral sulfation pathways, as documented by profound serum dehydroepiandrosterone sulfate (DHEA-S) depletion to baseline levels of 3.15 µg/dL and 1.79 µg/dL, respectively. Strikingly, our results confirm that this complete deficit in the circulating DHEA-S pool exists alongside completely normal baseline testosterone levels and an absolute absence of clinical hyperandrogenism, premature pubarche, or virilization signs in both children. These cumulative findings systematically map our patients within a tightly bounded clinical variant layer, establishing that biallelic enzymatic impairment at these specific residues produces an invariant, highly predictable skeletal phenotype presenting with variable endocrine expressivity.

## 4. Discussion

This study presents a comprehensive pathogenicity assessment of two homozygous missense variants in the *PAPSS2* gene [c.227T>A p.(Leu76Gln) and c.143C>G p.(Thr48Arg)] identified in two unrelated Turkish girls presenting with brachyolmia type 4 (BCYM4, OMIM 612847). Through integrated multi-modal analysis, both variants meet the joint consensus criteria for reclassification from variants of uncertain significance (VUS) to a definitive Pathogenic status [19]. Crucially, our conclusions do not stand as isolated in silico assertions; instead, they merge advanced structural characterization with established functional data to provide molecular-level insights into alterations disrupting the critical adenosine-5′-phosphosulfate (APS) kinase domain (residues 48–200) essential for normal PAPS biosynthesis [1,2,4].

The ultra-rare population status of both missense alleles is confirmed by their total absence in the gnomAD database (minor allele frequency < 0.00001), comfortably satisfying the ACMG moderate rarity criterion PM2. Strikingly, although the p.(Thr48Arg) variant was initially uncovered over 15 years ago [2]. It had never been rigorously characterized using contemporary computational standards prior to this investigation. The reidentification of these specific coordinates in the Turkish population expands the documented global mutational spectrum compiled in our comprehensive literature curation (The following supporting information can be downloaded at: https://www.mdpi.com/article/10.3390/medicina62071363/s1, Table S1), which details more than 92 cases across 18 countries spanning a wide phenotypic layout from severe spondyloepimetaphyseal dysplasia Pakistani type (SEMD-Pak) to mider brachyolmia subtypes [2,6,8,13].

To avoid internal predictive redundancy and statistical inflation during curation, our multi-modal computational framework was evaluated strictly as a single, interdependent line of evidence under the supporting ACMG pathogenicity category PP3. AlphaMissense, leveraging the deep learning architectures of AlphaFold2, assigned definitive pathogenic probability scores of 0.92 for p.(Leu76Gln) and 0.96 for p.(Thr48Arg). This consensus is mirrored by the REVEL ensemble method, which achieved a score of 0.85 [23], and Phred-scaled CADD metrics (28.8–29.1) that rank both substitutions within the top 0.1% most deleterious alterations in the human genome [24]. Rather than functioning as standalone proofs, these mathematical models strictly serve to illustrate the physical microenvironment consequences of alterations that impair the conserved catalytic domain, aligning with independent functional assays demonstrating that variants inside this locus yield a complete loss of enzymatic activity [1,11,25].

Three-dimensional structural modeling highlights the distinct spatial pathways underlying this catalytic failure. The p.(Leu76Gln) variant replaces a buried, hydrophobic leucine residue with a polar glutamine, resulting in a radical Grantham score of 113 and a severe localized negative shift of −7.3 units on the Kyte-Doolittle scale [21]. This dramatic polarity shift provides quantitative evidence for the disruption of the protein’s internal hydrophobic core packing. Conversely, the p.(Thr48Arg) variant centers on active-site steric hindrance and electrostatic repulsion. Introducing a bulky, basic arginine side chain right next to the dynamic N-terminal ATP-binding pocket creates a localized mass gain (+55.1 Da) and positive charge that directly compromises substrate orientation parameters and ATP-binding capacity without collapsing the underlying core architecture [2].

The developmental origins of skeletal deformities in *PAPSS2* deficiency merit further consideration. De Maio et al. demonstrated that structural alterations occurring during prenatal skeletal development can exert lasting influences on postnatal bone growth and morphology, providing a conceptual framework applicable to the vertebral abnormalities observed in our patients [26]. Furthermore, Ippolito et al. documented that skeletal deformities in disorders of abnormal bone biology are not static phenomena but evolve dynamically throughout childhood and adolescence [27]. This principle has direct clinical relevance for *PAPSS2* deficiency: the platyspondyly and growth retardation observed in our prepubertal patients at ages 5–6 years may not represent the final phenotypic expression, and longitudinal orthopedic surveillance through skeletal maturity is therefore strongly recommended.

This stringent constraint is further supported by deep evolutionary conservation. Both the Thr48 and Leu76 loci exhibit 100% sequence identity across all seven evaluated vertebrate species, representing approximately 500 million years of evolutionary constraint and satisfying the moderate criterion PM1. This absolute invariance indicates that these residues are under maximal purifying selection pressure. This principle is clinically mirrored by the uniform presentation of our Turkish probands, who exhibit the classic features of the BCYM4 phenotype—including short trunk disproportionate short stature, delayed carpal skeletal age, rectangular vertebral body flattening (platyspondyly), and irregular endplate sclerosis—consistent with the broader cohort data compiled by Helvacioglu and Guran [5] and the cross-population patterns recently described by Long and Luo [11].

Biochemically, both probands manifest a profound failure in peripheral sulfation pathways, showing severe depletion of circulating serum DHEA-S levels (3.15 µg/dL and 1.79 µg/dL) down to less than 2% of the lower normal limit. Remarkably, despite carrying the identical homozygous p.(Thr48Arg) variant, our prepubertal cases diverge dramatically from the historical index patient described by Noordam et al., who presented at 14.5 years of age with clinical hyperandrogenism, premature pubarche, and a severe polycystic ovary syndrome (PCOS)-like phenotype [2]. Our 5-to-6-year-old patients maintain completely normal-to-low circulating testosterone pools alongside a total absence of virilization or premature adrenarche signs. This prominent endocrine divergence supports cohort tracking data confirming that structural skeletal dysplasia is an invariant, universal hallmark of *PAPSS2* deficiency, whereas downstream androgen excess manifests in fewer than 5% of cases worldwide [5]. This biochemical dissociation implies that *PAPSS2* loss of function constitutes a necessary but insufficient condition for adrenal androgenization, suggesting that the phenotype may heavily depend on pubertal developmental stages or individual physiological steroidogenic variations.

Both families exhibit consanguinity, which aligns perfectly with the known epidemiology of autosomal recessive *PAPSS2* deficiency, where consanguineous unions are documented in over 60% of reported kindreds globally [1,4,9,10,15,16,28]. For these affected families, genetic counseling must emphasize the classic 25% recurrence risk for future pregnancies, where prenatal genetic testing via chorionic villus sampling (CVS) at 10–15 weeks gestation enables early detection and informed reproductive decision-making, as validated by Biancotto et al. and Handa et al. [7,8].

Ultimately, the integration of all lines of evidence within the joint 2015 ACMG/AMP framework confirms a Pathogenic status. Most importantly, independent cell–based in vitro enzymatic assays cross-referencing these exact catalytic positions provide the strong biological proof of direct functional loss required to satisfy criterion PS3 [2,25]. When combined with critical domain mapping (PM1), population rarity (PM2), multi-generational family co-segregation (PP1), single-layer computational consensus (PP3), and highly specific phenotype matching (PP4), this framework allows a confident and transparent classification that transforms uncharacterized VUS into clinically actionable markers [20].

### 4.1. Limitations

Several limitations of this study warrant acknowledgment. First, de novo in vitro functional assays were not performed directly within this project, leaving our classification dependent on published functional validation and computational consensus models [2,25]. Second, the current childhood window of the probands (ages 5–6 years) prevents a longitudinal view of adult skeletal progression or future pubertal endocrine developments. Third, the localized mechanistic basis for severe DHEA-S depletion without compensatory androgen excess remains unexplained, as omics-based investigation of potential modifier loci was not undertaken. Fourth, the two-case single-center design limits broad generalizability across diverse ethnic backgrounds. Fifth, structural assessments rely on machine learning-derived AlphaFold2 models rather than experimentally resolved human crystal configurations. Sixth, molecular dynamics simulations were not performed; structural analyses therefore rely exclusively on static AlphaFold2 predictions, which do not capture conformational flexibility or dynamic substrate interactions. Eighth, AI-based pathogenicity prediction tools carry inherent limitations, including potential training data biases and the risk of circular reasoning when multiple tools share underlying datasets; these caveats should be considered when interpreting the computational evidence presented. Finally, Middle Eastern and South Asian allele tracking remains underrepresented in global reference databases like gnomAD, making population rarity parameters provisional pending the growth of large, regional genetic registries.

### 4.2. Future Directions

Future initiatives should prioritize a prospective international registry with standardized clinical and skeletal reporting protocols coordinated through the Global Rare Bone Diseases network to clarify structure-function conclusions. Replicating these specific substitutions in transfected HEK293T/H295R cell systems to perform targeted PAPS quantification would provide direct biochemical corroboration of our structural predictions. Importantly, effective implementation of such a registry will require robust data management infrastructure. Innovative approaches including blockchain-based interoperable registry frameworks may facilitate secure multicenter sharing of longitudinal clinical, radiological, biochemical, and genomic data essential for rare skeletal disorders such as PAPSS2 deficiency [29]. Furthermore, longitudinal endocrine monitoring of the probands through adolescence will be crucial to clarify the impact of chronological developmental stages on the potential emergence of latent androgen excess. Standardized radiological surveillance and prospective growth evaluation remain the primary clinical pathways required to characterize adult skeletal morbidity and optimize early pediatric interventions.

## 5. Conclusions

In conclusion, this study provides a detailed structural and computational pathogenicity evaluation of two homozygous *PAPSS2* variants [novel c.227T>A p.(Leu76Gln) and rare c.143C>G p.(Thr48Arg)] identified in two unrelated Turkish girls presenting with brachyolmia type 4. By integrating current computational consensus tools with structural prediction models within the 2015 ACMG/AMP framework, both variants fulfill the parameters for reclassification as Pathogenic. Structural characterization suggests distinct mechanistic pathways: p.(Leu76Gln)-mediated hydrophobic core destabilization versus p.(Thr48Arg)-mediated active-site steric hindrance, both of which are predicted to compromise catalytic capability based on established domain constraints. The clinical layout—characterized by BCYM4 skeletal dysplasia paired with profound DHEA-S depletion and normal baseline androgen pools—aligns with the contemporary understanding of *PAPSS2* deficiency as a primary skeletal phenotype presenting with variable endocrine manifestations.

Future research priorities should focus on expanding international registry data to further clarify long-term genotype-phenotype correlations and disease heterogeneity across diverse age groups. Immediate clinical milestones include continuous longitudinal endocrine monitoring of prepubertal patients and direct de novo functional characterization through cell-based expression assays. Resolving these molecular mechanism coordinates remains essential to optimize early pediatric interventions, refine diagnostic workflows, and ensure precise genetic counseling for affected families.

## Figures and Tables

**Table 1:** Patogenicity of detected variants.

*GENE*	*PAPSS2*	
*TRANSKRIPT ID*	*NM_004670.4*	
*DBSNP*	*Novel*	*rs121908951*
*VARIANT*	*c.227T>A p.(Leu76Gln)*	*c.143C>G p.(Thr48Arg)*
*VARIANT LOCUS*	*Chr10-89472913 Exon 3*	*Chr10- 89469068*
*VARIANT TYPE*	*Missense*	*Missense*
*AGGREGATED PREDICTION*	*Deleterious (0.88)*	*Deleterious (0.88)*
*MUTATION ASSESSOR*	*Hi (4.88)*	*Hi (4.91)*
*SıFT*	*Deleterious*	*Deleterious*
*GERP*	*Uncertain (5.87)*	*Uncertain (5.46)*
*REVEL*	*Deleterious (0.92)*	*Deleterious (0.91)*
*DANN*	*Deleterious (1)*	*Deleterious (0.99)*
*PRIMATEAI*	*Deleterious (0.84)*	*Uncertain (0.77)*
*SEQ.GENOMIZE*	*Not found*	*Not found*
*CLINVAR*	*Not found*	*Pathogenic (1)*
*AFFECTED DOMAINS*	*Adenylylsulphate kinase*	*Adenylylsulphate kinase*
*CONSERVATION*	*Conserved*	*Conserved*
*ACMG CLASSIFICATION*	*Variant of uncertain significance*	*Variant of uncertain significance*
*ACMG PATHOGENICITY CRITERIA*	*PM2,PP3,PP5*	*PM2,PP3,PP5*

**ACMG:** The American College of Medical Genetics and Genomics; **Meta Score Pathogenicity Algorithms:** REVEL, aggregated prediction; **Individual Pathogenicity Algorithms:** Mutation assessor, SIFT, GERP, DANN, PrimateAI; **gnomAD**: Genome Aggregation Database; **Seq.genomize:** web-based annotation programme-Turkish population variant database.

**Table 2:** Summary of in silico prediction scores and reclassification of *PAPSS2* variants.

Protein Change	cDNA Change	Domain	gnomAD Frequence	REVEL Score	CADD(Phred)	AlphaMissense	New Classification
p.(Leu76Gln)	c.227T>A	APS-Kinase	-	0.85	29.1	0.92	Likely Pathogenic
p.(Thr48Arg	c.143C>G	APS-Kinase	-	0.85	28.8	0.96	Likely Pathogenic

**Table 3:** Quantitative physicochemical profiling of the identified *PAPSS2* variants.

Variant	Grantham Score	Hydropathicity Change (Δ)	Predicted Structural Impact
**p.(Leu76Gln)**	113	−7.3	**Destabilizing:** Severe hydrophobic core collapse
**p.(Thr48Arg)**	71	−3.8	**Destabilizing:** Steric hindrance and loss of stability

Delta (Δ): Represents the net change in the hydropathicity index calculated via the standard Kyte-Doolittle scale (Δ = Score of mutant protein-Score of wild protein). A negative value denotes a localized shift toward hydrophilicity, highlighting structural destabilization within the critical catalytic domain core.

## Data Availability

De-identified sequencing analyses and bioinformatic outputs (e.g., variant call/annotation reports, STRING network files, and structural modeling outputs) that support the findings of this study are available from the corresponding author upon reasonable request and subject to ethical approval.

## Supplementary Materials

The following supporting information can be downloaded at: https://www.mdpi.com/article/10.3390/medicina62071363/s1, Table S1: Comprehensive Literature Curation of Published PAPSS2 Variants and Clinical Phenotypes (1998–2026).
