# Supplementary material for: Reclassification of PAPSS2 Missense Variants in Turkish Patients with Brachyolmia Type 4: Multi-Modal Computational and Structural Biology Evidence for APS-Kinase Domain Dysfunction
Source: Medicina (Kaunas). 2026 Jul 15;62(7):1363. doi: 10.3390/medicina62071363 (PMC13413912; doi:10.3390/medicina62071363)
Supplement: Supplementary file 1 [file medicina-62-01363-s001.zip › medicina-4365994-supplementary.pdf]

Supplementary Table S1. Comprehensive Literature Curation Of Published PAPSS2 Variants And Clinical Phenotypes (1998–2026)

Year	Authors	Country/Region	PAPSS2 Variant(s)	Variant Type	N	Gender	Age at Diagnosis	Clinical Phenotype	Miscellaneous Findings
**1998**	Ahmad et al. [4]	Pakistan	c.1313C>A (p.Ser438Ter)	Nonsense	16	11M, 5F	Birth–adult	SEMD-Pak: short stature, bowed legs, enlarged knees, normal intelligence	Delayed ossification; early-onset OA; distinctive skeletal dysplasia phenotype
**2009**	Noordam et al. [2]	Germany/Turkey	c.143C>G p.(Thr48Arg) + c.985C>T p.(Arg329Ter)	Missense + Nonsense	1	F	14.5 years	Premature pubarche, PCOS-like, short stature, low DHEAS	High androgens; T48R in APS kinase; monogenic androgen excess
**2012**	Miyake et al. [6]	Turkey, Japan, Korea	c.337_338insG p.(A113GlyfsTer18) + 5 others	Frameshift/Deletion (6 types)	6	Mixed	5–18 years	Brachyolmia: short-trunk, spinal deformity	Platyspondyly, rectangular vertebrae, precocious rib calcification
**2013**	Iida et al. [1]	Japan, Turkey, Europe	p.(Cys43YTyr), p.(Leu76Gln), p.(Glu183Lys), p.(Val40Asp) (9 types)	9 types of variants	13	Mixed	Child–adolescent	PAPSS2-brachyolmia: short-trunk, broad PIP joints, spinal dysplasia	Platyspondyly, metaphyseal changes, rectangular vertebrae, Hobaek/Toledo types
**2013**	Tuysuz et al. [10]	Turkey	c.985C>T p.(Arg329Ter)	Nonsense	5	Mixed	2–21 years	SEMD-Pak expansion: short stature, kyphosis, low DHEAS	Precocious costal calcification, phenotype expansion

**2015**	Oostdijk et al. [28]	Netherlands/Europe	c.1371del + c.809G>A	Frameshift + Missense	2	M	Adolescent	Disproportionate short stature, DHEA challenge positive	PCOS-like in maternal heterozygous carrier
**2016**	Handa et al. [8]	Sweden, Japan, USA	Compound heterozygous variants	Mixed	1	M	Birth–10 years (prenatal)	AR-brachyolmia: early platyspondyly, dumbbell deformity	Progressive platyspondyly; femoral bowing→diminishes with age
**2019**	Bownass et al. (DDD Study) [13]	UK, Europe, USA, Canada, Australia	c.809G>A + 9 novel variants	Mixed (9 novel)	18	Mixed	Infancy–19 years	AR-brachyolmia: short spine, pain/stiffness variable, low DHEAS	Short femora prenatally; short-spine emerges in childhood
**2019**	Eltan et al. [15]	Turkey	c.1097-2A>G (novel deletion)	Large frameshift deletion	1	F	7.5 years	Short stature, premature pubarche, skeletal dysplasia, low DHEAS	Platyspondyly; 3rd premature pubarche case; first large deletion
**2021**	Perez-Garcia et al. [9]	USA (Jordanian)	c.1487A>C (H496P)	Missense (novel)	2	1F, 1M	F: 10→21 yr; M: 21 mo→15 yr	Brachyolmia, disproportionate short stature, low DHEAS	Consanguineous; normal GH stimulation; sulfonation defect
**2022**	Mustafa et al. [15]	Pakistan	c.1037G>C p.(Arg346Pro)	Missense (novel)	1	M	~7 years	Brachyolmia Hobaek type, short trunk	Consanguineous; altered protein expression
**2022**	Cao et al. [14]	China	c.712C>T p.(Arg238Ter) [PAPSS2 in family 4]	Nonsense (reported)	1	M	Child	SEMD Pakistani type: short stature, brachyolmia, kyphosis	Co-identified with ACAN variants in 4 Chinese families

**2024**	Helvacioğlu & Güran [5]	Turkey (Review)	Comprehensive review (65 patients)	All types	65 (cumulative)	Mixed	All ages	Bone phenotype always present; androgen excess <4%	Systematic analysis of skeletal phenotypes; 4 bone phenotype categories
**2025**	Long & Luo [11]	China	Novel compound heterozygous variants	Compound heterozygous	1	M	Birth (prenatal; 2 yrs 9 mo diagnosis)	BCYM4: short-trunk, platyspondyly, GH therapy responsive	Birth length 45 cm (−3.11 SD); GH improved height −5.02→−3.87 SD
**2026**	**This Report**	**Turkey (Istanbul)**	**c.227T>A p.(Leu76Gln) + c.143C>G p.(Thr48Arg)**	**1 Novel + 1 Rare Missense**	**2**	**Both F**	**6 years & 5 years**	**BCYM4: short-trunk, platyspondyly, delayed skeletal maturation, DHEAS deficiency**	**DHEA-S: 3.15 & 1.79 µg/dL (severely low); normal androgens; consanguineous families (1st cousin parents)**

SEMD-Pak = Spondyloepimetaphyseal Dysplasia Pakistani type; BCYM4 = Brachyolmia Type 4 (OMIM 612847); AR = Autosomal Recessive; OA = Osteoarthritis; DHEAS = Dehydroepiandrosterone Sulfate; DHEA = Dehydroepiandrosterone; PCOS = Polycystic Ovary Syndrome; PIP = Proximal Interphalangeal; GH = Growth Hormone; DDD = Deciphering Developmental Disorders; N = Number of patients; M = Male; F = Female; yr = Year; mo = Month.

** This curated case repository encompasses >92 documented *PAPSS2*-deficiency cases from >18 countries (1998–2026), including

two novel cases from Turkey reported in this study.** *PAPSS2* variants produce a spectrum of skeletal dysplasias ranging from SEMD-Pak (severe bone involvement) to brachyolmia subtypes (Hobaek, Toledo, BCYM4), with variable androgen metabolism abnormalities. Bone phenotype is universal; androgen excess is rare despite low DHEAS. Consanguinity is significant risk factor. The two novel Turkish cases [p.(Leu76Gln and p.(Thr48Arg)] expand the *PAPSS2* variant spectrum and demonstrate severe DHEAS deficiency with disproportionate short stature phenotypes.
